# Podocyte Biology in Pediatric Glomerular Disease: From Slit Diaphragm Assembly to Genotype-Guided Therapy—A Narrative Review

**DOI:** 10.3390/life16101722

**Published:** 2026-10-15

**Authors:** Petar Todorović, Andrea Kopilaš, Anita Racetin, Mirko Maglica, Azer Rizikalo, Fila Raguž, Nela Kelam, Katarina Vukojević

**Affiliations:** 1 Department of Anatomy, Histology and Embryology, University of Split School of Medicine, 21000 Split, Croatia; petar.todorovic@mefst.hr (P.T.); anita.racetin@mefst.hr (A.R.); nela.kelam@mefst.hr (N.K.); 2 Health Centre of Split-Dalmatia County, 21000 Split, Croatia; andrea.kopilas@dz-sdz.hr; 3 Department of Anatomy, University of Mostar School of Medicine, 88000 Mostar, Bosnia and Herzegovina; mirko.maglica@mef.sum.ba (M.M.); azer.rizikalo@mef.sum.ba (A.R.); 4 Department of Urology, University Clinical Hospital Mostar, 88000 Mostar, Bosnia and Herzegovina; 5 Department of Nephrology, University Clinical Hospital Mostar, 88000 Mostar, Bosnia and Herzegovina; fila.raguz@mef.sum.ba

**Keywords:** podocyte, slit diaphragm, nephrin, steroid-resistant nephrotic syndrome, focal segmental glomerulosclerosis, pediatric chronic kidney disease, urinary biomarkers, anti-nephrin antibodies, kidney biopsy, narrative review

## Abstract

The podocyte is a terminally differentiated cell whose foot processes and slit diaphragm form the final layer of the glomerular filtration barrier. Because podocytes are not effectively replaced after birth, and nephron number is fixed before it, their cumulative loss converts congenital and steroid-resistant nephrotic syndrome, focal segmental glomerulosclerosis and Alport syndrome into a shared trajectory towards chronic kidney disease. This narrative review, based on a structured search of three databases with the cited evidence classified as pediatric, adult, mixed or preclinical, connects podocyte biology with pediatric nephrology. We follow maturation and the modules whose disruption produces disease, a genetic architecture in which diagnostic yield falls steeply with age at onset, and the convergence of genetic, mechanical, metabolic and immune insults on podocyte depletion. Single-cell and spatial atlases, organoids, glomerulus-on-a-chip systems and human tissue are compared as partly discordant platforms. A molecular result currently changes a small, defined set of clinical decisions. No podocyte-derived urinary marker has been shown in children to add information beyond proteinuria and filtration rate, anti-nephrin antibody assays are not yet standardized, and kidney biopsy remains the reference standard. We set out an integrated diagnostic pathway, state the limitations of the evidence and define research priorities.

## 1. Introduction

Chronic kidney disease in childhood is dominated by congenital and hereditary disorders rather than by the metabolic and vascular causes that prevail in adults, while the contribution of glomerular disease increases with age at presentation [1]. Idiopathic nephrotic syndrome is the most frequent pediatric glomerular disease, with a reported annual incidence of 1.15 to 16.9 cases per 100,000 children that varies substantially with ancestry and geography [2,3]. Across this clinically heterogeneous group, outcome converges on a single cellular endpoint.

The glomerular filtration barrier is conventionally described as three layers, but its components are not functionally equivalent. The fenestrated endothelium and the glomerular basement membrane (GBM) restrict macromolecular passage in a graded and partly redundant fashion, whereas the podocyte layer, consisting of interdigitating foot processes bridged by the slit diaphragm, constitutes the final and least replaceable element of the barrier. Podocytes are terminally differentiated and largely postmitotic, so their loss is cumulative rather than transient, and podocyte depletion has been causally linked both to the magnitude of proteinuria and to the development of glomerulosclerosis [4]. Two changes must be distinguished throughout what follows: foot process effacement, a reorganization of the podocyte actin cytoskeleton that can reverse, and podocyte depletion, the irreversible loss of cells from a population that is not replenished. They are not interchangeable, and the markers discussed in Section 9.1 report on different points of the sequence between them. In children a second constraint compounds the first: nephron number is fixed once nephrogenesis ends at approximately 36 weeks of gestation and does not increase thereafter, so prematurity and intrauterine growth restriction reduce the endowment against which every subsequent podocyte loss is measured [5].

The entities discussed in this review are defined as follows. Congenital nephrotic syndrome (CNS) denotes nephrotic-range proteinuria with hypoalbuminemia and edema manifesting in utero or within the first three months of life and is most often caused by genetic defects in podocyte proteins [6,7]. Steroid-resistant nephrotic syndrome (SRNS) is defined by failure to achieve complete remission after four to six weeks of adequate glucocorticoid therapy [8]. Focal segmental glomerulosclerosis (FSGS) is a histological pattern of injury rather than a disease entity and is the lesion most frequently underlying SRNS in children [3,8]. The COL4A3, COL4A4 and COL4A5 glomerulopathies, including classical Alport syndrome, are inherited disorders of the GBM collagen IV network that present with persistent hematuria and progressive proteinuria [9]. Secondary glomerulopathies are those in which podocyte injury is downstream of a systemic process, as in lupus nephritis or IgA vasculitis nephritis.

Two developments have reoriented the field. High-throughput sequencing established that a monogenic cause can be identified in a substantial minority of children with SRNS, with a diagnostic yield that is inversely related to age at onset and considerably higher in early-onset and consanguineous cohorts [8,10,11]. Independently, autoantibodies targeting nephrin were identified in patients with minimal change disease and in children with idiopathic nephrotic syndrome, where they track with disease activity, differ markedly in prevalence between steroid-sensitive and genetic disease, and reproduce a podocytopathy on passive transfer [12,13,14,15]; the assays used to detect them are not yet standardized (Section 9.1). In parallel, single-cell and spatial atlases of the human kidney have redefined the reference against which glomerular cell states are compared [16], while stem cell-derived organoids and microfluidic glomerulus models have made human podocyte biology experimentally tractable in ways that were unavailable when much of the foundational work was performed.

## 2. Methods

**Figure 1:**
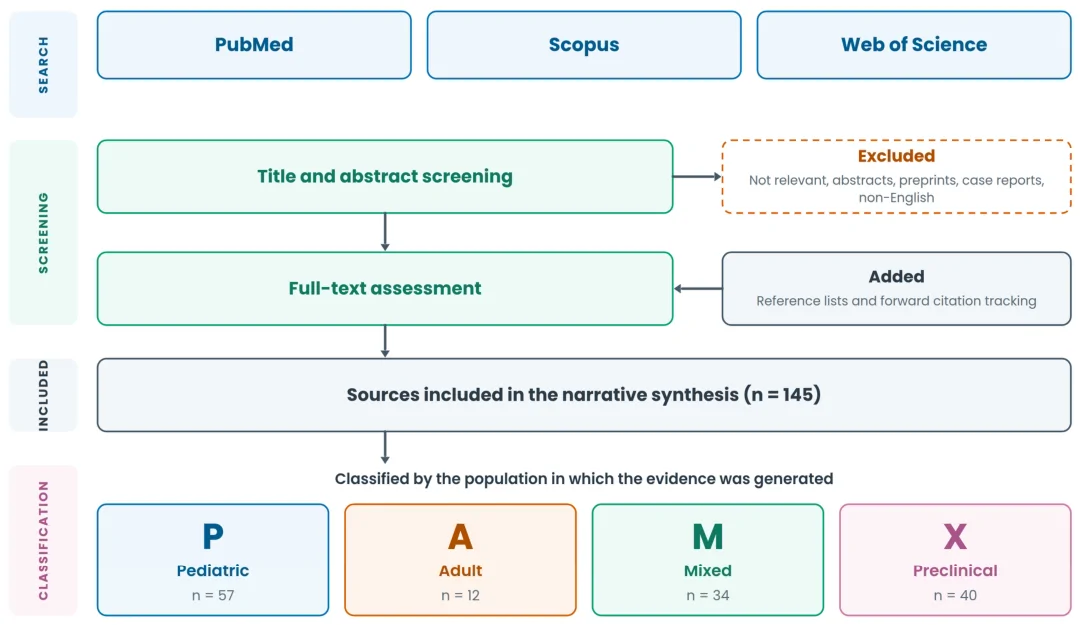
Literature search and study selection process. MEDLINE (PubMed), Scopus and the Web of Science Core Collection were searched from database inception to 14 April 2026, and targeted searches were updated during revision in September 2026. Records were screened by title and abstract and assessed in full text, and further records were added from reference lists and by forward citation tracking. Each of the 145 included sources was classified by the population in which its primary evidence was generated (P, pediatric human; A, adult human; M, mixed; X, preclinical), with two methodological standards left unclassified; the classification of every reference is given in Table S1. The figure is a schematic of the workflow and not a PRISMA flow diagram; counts of records identified, screened and excluded are not reported, consistent with the narrative review methodology.

This article is a narrative review, prepared with reference to the Scale for the Assessment of Narrative Review Articles (SANRA) [17]. A structured but non-systematic search was performed to identify the literature relevant to its scope. No protocol was registered, no formal risk-of-bias instrument was applied and no quantitative synthesis was attempted, which is consistent with the narrative format and with material that spans structural biology, developmental genetics, experimental modeling and clinical nephrology and is not reducible to a common effect measure. The search and selection process is summarized in Figure 1.

### 2.1. Search Strategy

MEDLINE (PubMed), Scopus and the Web of Science Core Collection were searched from database inception; the database searches were last performed on 14 April 2026, and the targeted searches and forward citation tracking described below were updated during revision of the manuscript in September 2026. Blocks of terms were combined with the Boolean operator AND. The first was structural: podocyte, slit diaphragm, foot process, nephrin, podocin, glomerular filtration barrier, glomerular basement membrane. The second was nosological: nephrotic syndrome, steroid-resistant nephrotic syndrome, congenital nephrotic syndrome, focal segmental glomerulosclerosis, Alport syndrome, podocytopathy, glomerulosclerosis, proteinuria. The third, applied to the clinically oriented sections, restricted by age: child, children, pediatric, paediatric, infant, neonate, adolescent. Targeted searches were then run for each gene listed in Table 1, for each experimental platform compared in Table 2 and for each candidate marker listed in Table 3. Reference lists of retrieved reviews, consensus statements and clinical practice recommendations were screened manually. Forward citation tracking was applied deliberately rather than incidentally in order to retrieve later reassessments of influential findings; this is how the reappraisal of soluble urokinase receptor [18], the failure to reproduce podocyte B7-1 staining [19] and the current methodological dispute over anti-nephrin antibody assays [20,21,22] were identified, and each is reported here alongside the original finding rather than in its place.

### 2.2. Study Selection

Peer-reviewed publications in English were eligible. Where several sources addressed the same question, priority was given, in order, to: clinical practice recommendations and consensus statements of IPNA, ERKNet, ESPN, ERA and KDIGO; pediatric human cohorts and disease registries; the original description of a gene–disease association or of a structural or mechanistic finding, irrespective of its date or of the system in which it was obtained; and the largest or most recent series addressing the question. Conference abstracts, preprints that had not completed peer review and single case reports were excluded, the last unless the report constituted the first description of a gene–disease association or of its mode of inheritance. No lower date limit was applied, because several foundational observations are cited specifically in order to document how they were later revised, including the 1974 porous-substructure model of the slit diaphragm [23] and the 1974 proposal that lipoid nephrosis is a disorder of T-cell function [24]. Titles and abstracts were screened by one author (P.T.), full texts of potentially eligible records were assessed against the criteria above, and the final set was reviewed by N.K. and K.V., with disagreements resolved by discussion. Selection was purposive rather than exhaustive; the consequences are considered in Section 11.

### 2.3. Evidence Appraisal and Classification

The pediatric literature is thin relative to the adult and preclinical literature addressing the same questions, and the imbalance is not uniform across this review. To prevent it from being obscured, each cited source has been classified by the population in which its primary evidence was generated: P, pediatric human, including human fetal and neonatal material; A, adult human; M, mixed pediatric and adult cohorts, and reviews, consensus statements or guidelines drawing on both; and X, preclinical, comprising animal models, induced pluripotent stem cell-derived organoids, microfluidic devices and immortalized cell lines. The classification of every reference is given in Table S1. Of the 145 sources cited, 57 are classified P, 12 A, 34 M and 40 X; the remaining two are methodological standards to which no population class applies. The distribution across the review is itself a finding: the account of molecular mechanism in Section 4, Section 5 and Section 7 rests predominantly on preclinical work, the genetic architecture in Section 6 rests predominantly on pediatric cohorts and registries, and the therapeutic evidence in Section 9.2 is mixed, with the newer agents tested only or mainly in adults. The labels are given for every reference in Table S1 and for every entry in Table 3 and Table 4. A finding obtained in an experimental system is not presented as applicable to children unless a pediatric human study is cited alongside it, and clinical results obtained only in adults are identified as such where they are cited.

For biomarkers and treatments, a second axis is used in addition to population, because sources at very different stages of validation would otherwise be discussed together. Established denotes a use supported by a clinical practice recommendation or by reproducible cohort data in children; emerging denotes a use supported by human data that include children, from multicenter or longitudinal studies, but that are not yet standardized or prospectively validated; and experimental denotes a use resting on preclinical work, on early-phase adult studies or on a single cross-sectional discovery series. A candidate whose original finding was not confirmed on reassessment, or that showed no added value when tested prospectively, is labeled not supported. Every entry in Table 3 and Table 4 is assigned to one of these categories, and the discussion of treatment in Section 9.2 is organized by them.

## 3. Podocyte Specification, Maturation and the Postmitotic Constraint

The number of podocytes a human kidney will ever possess is essentially fixed before birth. Nephrogenesis is complete at approximately 36 weeks of gestation, no new nephrons and therefore no new podocyte populations are generated afterwards, and the final nephron number varies widely between individuals and is reduced by preterm birth and intrauterine growth restriction [5]. Mature podocytes are in turn terminally differentiated and have permanently exited the cell cycle, so the loss of individual cells cannot be compensated for by the proliferation of their neighbors [25]. The oligonephropathy that follows an interrupted nephrogenesis has been estimated to affect between 8 and 24 per cent of preterm and low-birth-weight infants [26]. In a child these facts compound one another, because a reduced endowment lowers the total number of podocytes present while raising the filtration load borne by each glomerulus that was formed. Together they set the arithmetic of everything that follows: podocyte number can only fall, and the endowment it falls from was determined in utero.

**Figure 2:**
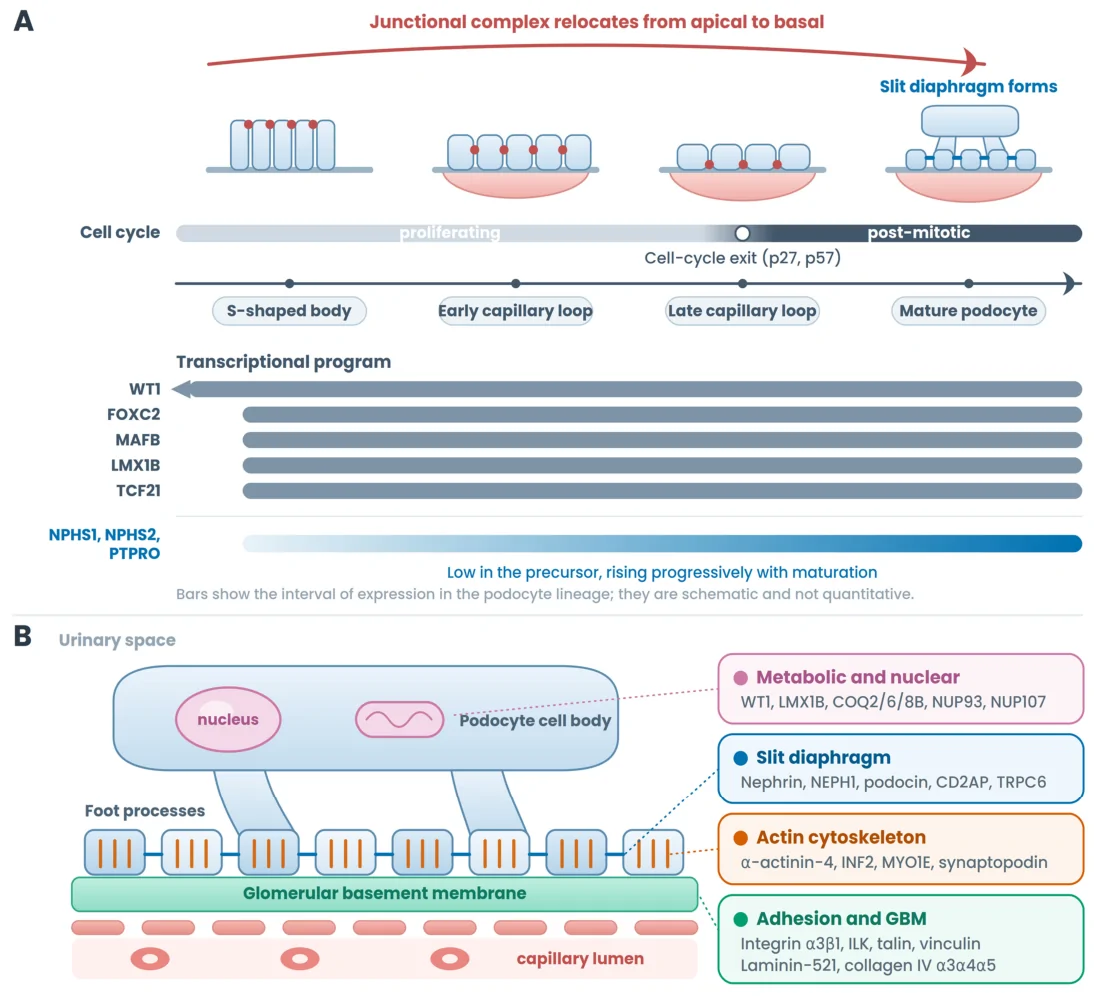
Podocyte maturation and the structural modules of the mature filtration barrier. (**A**) Stages of glomerular development from the S-shaped body to the mature capillary loop, showing the transcriptional program, relocation of the junctional complex from apical tight junctions to the basally positioned slit diaphragm, and exit from the cell cycle. Bars indicate the interval over which each factor is expressed in the podocyte lineage and are schematic rather than quantitative; the mature markers NPHS1, NPHS2 and PTPRO are low in the precursor and rise progressively with maturation. (**B**) Cross-section of the mature filtration barrier showing the slit diaphragm, actin cytoskeleton, adhesion and glomerular basement membrane, and metabolic and nuclear modules, with representative proteins for each. The module names correspond to the row groups of Table 1.

Podocytes arise within the nephron progenitor lineage during a stereotyped morphogenetic sequence. Progenitors condensed around the ureteric bud tip undergo mesenchymal to epithelial transition to form the renal vesicle, which folds into the comma-shaped and then the S-shaped body [26,27]. The lineage becomes morphologically identifiable at the comma-shaped stage, where internal folding separates a visceral epithelial layer that will become the podocytes from the parietal layer that will become Bowman’s capsule [26]. Within the S-shaped body these precursors occupy the visceral aspect of the proximal segment, and single-cell transcriptomics of the human fetal kidney identifies them as a discrete cluster expressing high levels of MAFB and FOXC2 but only low levels of the mature markers NPHS1, NPHS2 and PTPRO [28]. The transition to the capillary loop stage is where the cell acquires its defining architecture: the columnar epithelial cell flattens and arborises, the intercellular junctions migrate from an apical to a basal position, and the slit diaphragm replaces the tight junctions of the immature podocyte (Figure 2A). Nephrin transcripts are first detected in late S-shaped bodies of the human fetal kidney, and nephrin protein localizes to the earliest recognizable slit diaphragm regions [29]. Podocyte gene expression then continues to change progressively across gestational ages rather than switching at a single point [28], so maturity in this lineage is a gradient rather than a threshold. The S-shaped body is also the earliest stage at which insults impairing human nephrogenesis leave detectable traces, with the nephrogenic niche, pretubular aggregate and renal vesicle appearing to be spared [26].

A small transcriptional network establishes and then maintains this phenotype, but its members are not equivalent and their loss-of-function phenotypes differ sharply. Wt1 null mice fail to form kidneys at all, Foxc2 mutants form hypoplastic kidneys with few glomeruli and reduced Nphs2 expression, whereas Mafb, Tcf21 and Lmx1b mutants develop glomeruli whose podocytes are defective at the ultrastructural level [30]. Systematic elimination of the six podocyte transcription factors in the Xenopus pronephros showed that wt1 and foxc2 are together required, though not sufficient, for podocyte development, and that a third input, activated Notch signaling, was needed before podocyte genes could be induced ectopically [30]. The structural point matters more than the list: this is a combinatorial network rather than a linear cascade with a single master switch, which is why the individual factors sit at different levels and why the human phenotypes associated with them differ so markedly. Those phenotypes, from Denys–Drash and Frasier syndrome to nail–patella syndrome, arise from mutations in these same genes [30].

Terminal differentiation is then enforced by the cell cycle machinery itself. Mature podocytes abundantly express the cyclin-dependent kinase inhibitors p27 and p57, which hold the cell in quiescence by inhibiting cyclin–CDK complexes [31]. The constraint is not merely an inability to divide but an incompatibility between division and structure: because of its cytoskeletal organization the podocyte cannot maintain foot process architecture and complete cell division at the same time [31]. When injured podocytes are driven back into the cycle they undergo nuclear division without cytokinesis, generating binucleated and multinucleated cells that are more susceptible to injury and prone to detachment from the basement membrane or to apoptosis, an outcome termed mitotic catastrophe [31]. This is what distinguishes the podocyte from the tubular epithelium alongside it, where proliferation is a genuine repair response. In the podocyte, cell cycle re-entry is not repair but an additional route to loss.

Whether the mature glomerulus retains any capacity to replace podocytes remains contested. Parietal epithelial cells of Bowman’s capsule have been proposed as a local progenitor pool able to migrate onto the tuft and adopt podocyte markers, and harnessing this reserve is an active therapeutic aim, but the extent of genuine functional replacement in the adult human glomerulus is not established [4,25]. In practical terms the only adaptation available to the surviving cells is hypertrophy, which allows a smaller number of podocytes to cover an enlarging capillary surface for a time, though not without cost.

The transcriptional program outlined here converges on a single structure that both defines the mature podocyte and fails first in disease: the slit diaphragm.

## 4. The Slit Diaphragm as a Structural and Signaling Hub

The slit diaphragm is not a passive sieve but a signaling junction that continuously reports on its own mechanical state. For half a century its architecture was inferred from chemically fixed tissue. Perfusion fixation with tannic acid revealed a continuous junctional band with a zipper-like substructure and an apparently uniform population of rectangular pores, a model that implied a static, isoporous filter spanning the roughly 40 nm slit [23]. Cryo-electron microscopy of vitreous sections instead suggested a flexible, multilayered protein scaffold whose spacing was not fixed [32]. Cryo-electron tomography of high-pressure frozen native murine glomeruli has since resolved the junction as a fishnet: a quasi-crystalline, sheet-like polymer in which nephrin and NEPH1 cross one another through multiple homophilic and heterophilic contacts, an arrangement that confers stability and flexibility simultaneously [33]. The same bilayered, highly periodic architecture has been described in Drosophila nephrocytes [34] and, most recently, in native human glomeruli, where the mesh is densely interwoven [35]. This revision matters for the argument that follows, because a deformable polymer, unlike a fixed sieve, can transduce mechanical load.

Nephrin, encoded by NPHS1, supplies the bulk of that polymer (Figure 2B). Its ectodomain carries eight immunoglobulin-like domains and a single fibronectin type III module and engages nephrin molecules from the opposing foot process as well as NEPH1 in trans [36]. Its short cytoplasmic tail is the signaling element. Engagement of the ectodomain triggers transient activation of the Src family kinase Fyn, which phosphorylates conserved tyrosines falling into two groups with distinct binding preferences: phosphorylated YDxV motifs recruit the SH2 and SH3 adaptors Nck1 and Nck2, whereas other residues recruit phospholipase C isoforms and Crk family adaptors [36]. Nck1 and Nck2 are not interchangeable, and their combined loss disturbs actin bundling at focal adhesions and increases basement membrane matrix deposition [37]. The machinery that executes these instructions is the actin cytoskeleton; what belongs here is that the junction generates them.

Podocin, encoded by NPHS2, organizes the cytoplasmic face of the junction. It is a stomatin family protein anchored by a hairpin transmembrane segment with both termini in the cytosol, it accumulates in oligomeric form within the lipid rafts of the slit diaphragm at the insertion site of the junction, and it binds both nephrin and CD2AP through its carboxy-terminal domain [38]. Its role is now defined more precisely than as a generic scaffold: podocin provides regularly spaced intracellular anchor points that order the nephrin chains; pathogenic podocin variants abolish this ordering effect, and heterooligomers containing the common p.R229Q variant are associated with a wider spacing between neighboring nephrin molecules [39]. Podocin also binds cholesterol, and this is what allows it to regulate the activity of associated ion channels [40]. It therefore sits upstream of both the geometric spacing of the filter and its mechanical responsiveness, which explains why loss of podocin and loss of nephrin converge on similar phenotypes. The clinical consequences of individual podocin variants, including the trans-associated behavior of the common p.R229Q allele, follow from the same geometry [39,40].

The channel in question is TRPC6, a non-selective cation channel resident in the slit diaphragm complex whose architecture and pharmacological modulation have been resolved by cryo-electron microscopy [41]. TRPC6 in podocytes is gated both by diacylglycerol and by membrane stretch, and podocin has opposing effects on the two modes, suppressing stretch activation while enhancing the response to diacylglycerol [42]. The same assembly that spaces nephrin therefore also sets how the podocyte converts membrane tension into calcium entry. Affinity purification of native slit diaphragms shows that nephrin, NEPH1 and podocin form the core of a much larger co-assembled network of kinases, phosphatases, transporters, receptors and scaffolds, several of which are individually required for normal filtration [43]. That proteomic view supports reading the junction as a dynamically regulated sensor of filtration rather than as the final barrier [43]. Nephrin itself is continuously internalized and recycled, so the amount held at the junction reflects a balance between delivery and removal rather than a fixed pool [36]. NEPH1, FAT1, P-cadherin, CRB2 and the retained tight junction protein ZO-1 complete the junctional set.

Two observations tie these strands together. First, nephrin tyrosine phosphorylation is not a bystander mark. Clustering of nephrin generates measurable traction forces within minutes and activates paxillin, FAK and p130Cas, and abolishing phosphorylation at the YDxV motifs exacerbates proteinuria and accelerates podocyte detachment when glomerular mechanical stress is raised experimentally [44]. Second, in experimental podocyte injury the fall in nephrin phosphorylation precedes the onset of proteinuria and coincides with actin depolymerization [36]. The slit diaphragm therefore behaves as a sensor of its own loading state, and one of the earliest detectable events in podocyte injury is a change in the phosphorylation status of a junction that still appears structurally intact.

Nephrin signaling has no output, however, without the actin machinery it commands and no anchorage without the matrix beneath it.

## 5. Actin Cytoskeleton, Adhesion and the Podocyte–GBM Interface

Foot process architecture is an actin problem before it is a genetic one. The podocyte maintains three distinguishable cytoskeletal compartments: microtubules and intermediate filaments that give the cell body and the major processes their form, a contractile actomyosin system running along the length of the foot processes, and a branched cortical actin network beneath the plasma membrane [45]. The actomyosin system is not decorative: it generates the tension that opposes the distending force of glomerular capillary pressure, so this cytoskeleton is load-bearing as well as shape-determining [29,45]. Foot process effacement, the morphological change common to essentially all proteinuric disease, is a reorganization of these compartments rather than a structural collapse. The interdigitating processes retract, the contractile apparatus is remodeled, and in acute experimental injury the change can reverse [45]. Defining effacement this way matters for the rest of the review: it separates what electron microscopy shows, which is an actin phenotype, from podocyte loss, which is not, and it explains why insults as different as a monogenic cytoskeletal defect and a circulating factor produce the same picture [45].

Several actin-associated proteins are individually necessary for that architecture (Figure 2B). Alpha-actinin-4, encoded by ACTN4, crosslinks and bundles filamentous actin, and the point mutations that cause autosomal dominant FSGS increase rather than abolish its affinity for filamentous actin [46]. Both increased affinity and absence of the protein produce disease, so what the podocyte requires is a crosslinker of correctly tuned strength rather than simply the presence of one [45]. INF2 encodes a formin, a class of protein that nucleates and remodels actin filaments, and INF2 variants are a major cause of autosomal dominant FSGS with onset mainly in adolescence or adult life [47]. MYO1E encodes a non-muscle class I myosin that couples membranes to actin, and its biallelic loss causes childhood familial FSGS [48]. Anillin, encoded by ANLN, is a filamentous actin-binding protein whose mutation likewise causes FSGS [49]. ARHGDIA encodes a Rho GDP dissociation inhibitor whose loss deregulates Rho GTPase signaling directly [45]. The direction of the defect varies while its target does not: mistuned crosslinking, a lost motor and a lost GTPase brake all converge on the same foot process phenotype.

The Rho GTPases RhoA, Rac1 and Cdc42 set the operating point of this system, and the podocyte is unusual in that both excess and deficiency of the same GTPase are damaging: constitutively active and dominant negative RhoA each produce effacement and proteinuria in vivo, as does Rac1 activation [45]. Podocyte-specific deletion of the individual GTPases separates them, however, and only loss of Cdc42 abolishes foot process formation outright, which places Cdc42 in a different category from the other two [45]. Synaptopodin, an actin-associated protein of the foot process, stabilizes RhoA and with it the contractile architecture, and its degradation is one route by which calcium signaling is converted into cytoskeletal change [45].

None of this holds without anchorage. Foot processes attach to the GBM through integrin alpha3beta1, dystroglycan and discoidin domain receptor 1, and the integrin cytoplasmic tail is coupled to the actin network through talin, vinculin, paxillin and integrin-linked kinase, forming focal adhesion complexes that are the second signal-integrating hub of the foot process alongside the slit diaphragm [45,50]. These are not passive anchors. Clustering of nephrin activates paxillin, FAK and p130Cas within minutes [44], and the Nck adaptors that transmit slit diaphragm signals also organize actin bundling at focal adhesions through alpha-actinin-4 [37]. The apical junction and the basal adhesion therefore regulate one and the same actin network from opposite ends of the foot process, which is why a defect in either produces the same morphology. Mutations in these components cause human disease in their own right.

The matrix on the far side of that adhesion is itself specialized. The mature GBM arises by fusion of the podocyte-derived and endothelium-derived basement membranes and consists principally of laminin-521, the alpha3alpha4alpha5 network of collagen IV, nidogens and the heparan sulfate proteoglycan agrin, together with perlecan and minor components of restricted tissue distribution [51]. Agrin is the major heparan sulfate proteoglycan of the mature GBM, is expressed by podocytes, and tethers laminins to cell surface receptors [51]. Its composition is not fixed during development. The laminin of the earliest GBM, LM-111, is replaced through LM-511 by LM-521, and the early alpha1alpha1alpha2 collagen IV network is replaced by alpha3alpha4alpha5, which in the mature GBM is a product of the podocyte alone [50]. Laminin-521 is a ligand for integrin alpha3beta1, and intravenously delivered recombinant LM-521 incorporates stably and in the correct orientation into the GBM of laminin beta2-deficient mice, attenuating podocyte injury and foot process effacement and delaying proteinuria without preventing nephrotic syndrome [52]. The likely reason it falls short is itself informative: the injected trimer accumulated primarily on the endothelial aspect of the GBM, and its absence from the podocyte side was proposed as the explanation, which suggests that the GBM is an internally structured matrix rather than a homogeneous gel [52]. The GBM is thus at once the substrate the podocyte adheres to and the mechanical environment in which it works.

Each of the three modules described so far is defined clinically by the mutations that disrupt it, and in children those mutations follow a strikingly age-dependent distribution.

## 6. Genetic Architecture and Genotype–Phenotype Correlation in Pediatric Podocytopathies

In children, the probability that nephrotic syndrome is monogenic is largely a function of the age at which it presents. Among 1783 families with SRNS manifesting before 25 years of age a single-gene cause was found in 29.5% overall, but in 69.4% presenting in the first three months of life, 25.3% between one and six years and 10.8% between thirteen and eighteen years [53]. The PodoNet registry of 1655 children reproduces the gradient, from 66% in congenital disease to 15% to 16% in schoolchildren and adolescents [54]. Yield rises in consanguineous families [11,55] and falls once the earliest age group is excluded and the phenotype is defined strictly: in a series limited to onset after the first year, with hypoalbuminemia and no remission after four weeks of steroid monotherapy, monogenic variants were detected in 12% of 185 patients [56]. The gradient is a property of the population tested as much as of the disease itself.

The genes behind it are not distributed at random along it. Congenital and infantile disease is dominated by recessive defects of the junctional module, principally NPHS1 and NPHS2, with smaller contributions from PLCE1, LAMB2 and CRB2 and from de novo dominant WT1 variants [7,54,57]. Recessive disease predominates through later childhood, where NPHS2 remains the commonest single cause and MYO1E adds a further share [48], while the dominant genes ACTN4, INF2 and TRPC6 become relatively more prominent as onset moves into adolescence and adult life [53,58]. Table 1 gives the genes, modules, inheritance and typical onset; what it cannot convey is that age at presentation is itself the strongest single predictor of which module will be implicated and, consequently, of how a panel result should be read.

A substantial minority of monogenic cases are syndromic, and here the genotype discloses extrarenal risk the renal phenotype does not. WT1 shows this most sharply. In the PodoNet WT1 cohort of 61 patients, missense substitutions affecting DNA-binding residues were associated with diffuse mesangial sclerosis in 74%, with early onset and rapid progression to kidney failure, while sex reversal or urogenital anomalies (52%), Wilms tumor (38%) and gonadoblastoma (5%) were almost exclusive to WT1 disease [59]; national cohort data have since added late-onset glomerulopathy and cases where Wilms tumor is the only manifestation [60]. LAMB2 (Pierson syndrome), LMX1B (nail–patella syndrome) and SMARCAL1 (Schimke immuno-osseous dysplasia) behave comparably: in the largest SMARCAL1 cohort 11 of 34 patients were identified unexpectedly by SRNS panel testing rather than by phenotype, and those recognized clinically more often suffered cerebral ischemia and death before ten years [61]. Panels detect syndromic disease before the syndrome declares itself. In Galloway–Mowat syndrome, recessive variants in OSGEP, TP53RK, TPRKB and LAGE3, the four KEOPS complex subunits, were found in 37 individuals from 32 families besides the previously known WDR73, where the shared mechanism is impaired translation with endoplasmic reticulum stress and disordered actin regulation [62].

Two gene groups have no counterpart among the structural modules and are defined instead by mechanism. In the coenzyme Q10 biosynthesis pathway CoQ10 acts both as the electron carrier of the respiratory chain and as a lipid-soluble antioxidant, so loss of COQ2, COQ6, COQ8B or PDSS2 exposes the podocyte to oxidative and mitochondrial injury rather than to a structural defect. It explains 1% to 2.7% of SRNS and up to 10% of solved cases, and its three principal genes separate by age and extrarenal burden: across 251 patients kidney disease was first diagnosed at a median of 1.0, 1.2 and 9.8 years for COQ2, COQ6 and COQ8B, and was confined to the kidney at diagnosis in 34%, 10.8% and 70.7% [63]. The second group encodes nucleoporins. Variants in the inner ring component NUP93 disrupt pore assembly and abolish its interaction with SMAD4, interrupting BMP7 signaling, while a NUP205 variant abrogates the interaction with NUP93 [64]; some variants in the outer ring components NUP107, NUP85, NUP133 and NUP160 weaken contacts between adjacent subunits, and knockout of NUP107, NUP85 or NUP133 in podocytes activates Cdc42 [65], the GTPase whose loss abolishes foot process formation. A nuclear transport defect converges on the same cytoskeletal endpoint as a mistuned crosslinker.

The COL4A3, COL4A4 and COL4A5 genes are the largest single genetic contributor and the genes most often misassigned in children. About half the pathogenic variants in each gene are large rearrangements, truncating or splicing changes associated with earlier kidney failure, and with hearing and ocular involvement in X-linked males and in autosomal recessive disease; the rest are missense changes, mostly glycine substitutions, whose severity depends on the substituting residue and on the distance from a non-collagenous interruption. Heterozygous COL4A3 or COL4A4 variants, long associated with thin basement membrane nephropathy, are now classified as autosomal dominant Alport syndrome [66]. Because a collagen IV defect can produce nephrotic-range proteinuria with an FSGS lesion on light microscopy, rare COL4A3 or COL4A4 variants were found in seven of 70 families carrying a diagnosis of hereditary FSGS [67]. Extended sequencing with reverse phenotyping identified podocytopathy gene variants in 30% of 64 patients with SRNS and phenocopy genes, including COL4A3, COL4A5, CLCN5, GLA and PAX2, in a further 28% already treated as SRNS [68], whereas a whole-exome series of 300 families returned phenocopies in 3.7% [55]. That gap reflects panel breadth and referral pattern rather than biology.

Two loci act as modifiers rather than causes. NPHS2 p.R229Q is pathogenic only in trans with particular variants that alter podocin oligomerization, and is benign in the homozygous state and with other trans partners [69]; more than 15% of the p.R229Q associations reported in patients are benign, so its high allele frequency makes incidental detection in an affected child a genuine hazard [70]. APOL1 high-risk genotypes in African American children are associated with FSGS and faster progression [71], but pediatric data are sparse and self-reported ancestry is a poor proxy: in an admixed Brazilian pediatric cohort, high-risk genotypes were found in 8% of families, and three of the eight carriers self-identified as White [72].

A genetic result changes management in a few defined ways: withdrawal of immunosuppression in most monogenic forms, CoQ10 supplementation in the biosynthesis pathway, tumor and gonadal surveillance in WT1 disease, the timing of renin–angiotensin blockade in collagen IV disease, and a revised transplant risk, recurrence having occurred in 4.5% of children with a genetic diagnosis against 25.8% of those without [54].

Three practical constraints qualify all of the above, and they operate in the clinic rather than in the literature. The first is access. Comprehensive panel analysis of the known podocytopathy genes is the most cost-effective approach and is recommended in all children with primary SRNS [8], but a panel of 40 to 50 genes has been costed at approximately US$1000 to US$2200 with a turnaround of three to six weeks [73], and neither figure is uniform between health systems. A KDIGO controversies conference stated plainly that testing is often not affordable for patients or for health systems, and that access can remain unequal even where it is reimbursed [74]. Turnaround interacts directly with treatment: where the result will not be available within a few weeks, a calcineurin inhibitor is started at the end of the confirmation period and the decision revisited when the result arrives [8].

The second is interpretation. Variants are classified against the criteria of the American College of Medical Genetics and Genomics and the Association for Molecular Pathology [75], and a variant of uncertain significance is not a diagnosis: it neither licenses the withdrawal of immunosuppression nor excludes a monogenic cause, and it requires segregation analysis, deeper phenotyping or reanalysis as classification evidence accumulates [74]. Reverse phenotyping illustrates both the gain and the hazard: in the series described above, more than a quarter of the patients managed as having SRNS carried variants in phenocopy genes [68]. In a predominantly adult cohort of 3315 patients with chronic kidney disease, the molecular diagnosis reclassified the clinical diagnosis in about one in nine of the diagnosed patients for whom detailed data were available [76]. *NPHS2* p.R229Q, discussed above, remains the clearest pediatric example of a variant whose reporting can mislead [69,70].

The third is counseling, and the handling of information the family did not seek. Every guideline that recommends testing recommends counseling alongside it [7,8,9], and the reason is substantive rather than procedural: a *WT1* result carries tumor risk and a decision about gonadectomy [59], a collagen IV result changes how relatives and potential living donors are assessed [9], and an *APOL1* genotype carries ancestry information that self-report predicts poorly [72]. Actionable secondary findings arose in 1.6% of participants in the largest kidney exome series [76], and no consensus yet governs the reporting of incidental carrier status [74]. Where a clinical geneticist or genetic counselor is not available to the nephrology service, these are the components of genotype-guided care that are missing first, which matters most in early-onset and consanguineous cohorts, where diagnostic yield is highest [11,55].

Yet most children with podocyte injury carry no causative variant, and even those who do progress through a stereotyped set of downstream responses shared with acquired disease.

**Table 1:** Genes causing pediatric podocytopathies, grouped by the module they disrupt. References supporting each row follow the gene symbol. Typical onset is the age band of most frequent first presentation, with a published median or range where one exists. Management consequences are marked **E** where established, meaning supported by a clinical practice recommendation, by reproducible cohort data or by standard care of the associated extrarenal disease, and **I** where investigational, meaning based on isolated reports. A dash indicates that none is reported (extrarenal features) or established (management).

Gene (Protein) [Ref.]	Inheritance	Typical Onset	Renal Phenotype	Extrarenal Features	Management
**Slit diaphragm and junctional complex**					
*NPHS1* (nephrin) [6,7]	AR	Congenital	CNS	–	**E** No immunosuppression; anti-nephrin-mediated disease after transplantation
*NPHS2* (podocin) [54,69,70]	AR	Congenital–childhood	CNS, FSGS	–	**E** No immunosuppression; check trans allele
*PLCE1* [77]	AR	Congenital–infantile	DMS, FSGS	–	**I** Occasional response to immunosuppression
*CD2AP* [78,79]	AR	Infancy–adolescence ^a^	FSGS	–	–
*TRPC6* [80,81]	AD	Adult (median 24 y) ^b^	FSGS	–	–
*CRB2* [57,82]	AR	Prenatal–childhood ^c^	CNS, FSGS	Ventriculomegaly, raised AFP ^c^	–
*PTPRO* [83]	AR	Childhood (5–14 y)	FSGS, MCD	–	–
**Actin cytoskeleton**					
*ACTN4* [46]	AD	Early adulthood ^b^	FSGS	–	–
*INF2* [47,84]	AD	Adolescent–adult	FSGS	CMT neuropathy	**E** Neurological review
*MYO1E* [48]	AR	Childhood (1–9 y)	FSGS	–	–
*ANLN* [49]	AD	Not reported ^d^	FSGS	–	–
*ARHGDIA* [85]	AR	Congenital–infantile	DMS, FSGS	Neurological (some)	–
**Adhesion and glomerular basement membrane**					
*ITGA3* [86]	AR	Neonatal	CNS	Lung disease, epidermolysis bullosa	**E** Respiratory and skin care
*LAMB2* [6,87]	AR	Congenital	CNS, DMS	Microcoria (Pierson)	**E** Ophthalmology; no immunosuppression
*LAMA5* [88]	AR	Infantile–early childhood ^e^	FSGS	–	–
*COL4A3*, *COL4A4* [9,66,67]	AR, AD	Childhood–adult	Alport, FSGS	Deafness, ocular (AR)	**E** RAS blockade ^f^; genotype related donors
*COL4A5* [9,89]	XL	Childhood (males) ^g^	Alport	Deafness, lenticonus	**E** RAS blockade ^f^
**Metabolic and nuclear**					
*WT1* [59,60]	AD, de novo	Congenital–adolescent	DMS, FSGS	DSD, Wilms tumor, gonadoblastoma	**E** Tumor and gonadal surveillance
*LMX1B* [90,91]	AD	Childhood–adult	FSGS	Nail–patella dysplasia, glaucoma	**E** Glaucoma screening
*PAX2* [92,93]	AD	Childhood–adult	FSGS, CAKUT	Ocular anomalies	**E** Ophthalmology; urinary tract imaging
*SMARCAL1* [61]	AR	Childhood (median 4.5 y)	FSGS, MCD	Short stature, T-cell defect, stroke	**E** Immune and stroke surveillance
*COQ2*, *COQ6* [63,94]	AR	Infantile (median 1.0 and 1.2 y)	FSGS	Encephalopathy, retinopathy (*COQ2*); deafness (*COQ6*)	**E** CoQ10, started early
*COQ8B* (ADCK4) [63,94]	AR	School age (median 9.8 y)	FSGS	Usually none	**E** CoQ10 (benefit unproven)
*PDSS2* [95]	AR	Infantile	NS ^h^	Leigh syndrome	**E** CoQ10
*NUP93*, *NUP107* [64,65,96,97]	AR	Childhood ^i^	FSGS, DMS	Microcephaly (*NUP107*)	–
*OSGEP* (KEOPS), *WDR73* [62,98]	AR	Congenital–infantile	FSGS	Microcephaly (GAMOS)	**E** Prognostic counseling
*SGPL1* [99]	AR	Congenital–infantile	FSGS	Adrenal insufficiency, ichthyosis	**E** Adrenal replacement
*MT-TL1* [100,101]	Mt	Adolescent–adult	FSGS	Deafness, diabetes, MELAS	**E** Audiology; maternal inheritance counseling

^a^ Biallelic variants reported in few families, with onset between 10 months and adolescence. ^b^ Pediatric onset reported. ^c^ Congenital form detected prenatally with ventriculomegaly and raised AFP; later-onset disease presents as FSGS without these features. ^d^ Affected individuals described without a cohort-level age distribution. ^e^ Reported as variants of uncertain significance. ^f^ From 2 years once hematuria is present in males with X-linked and in all patients with autosomal recessive disease, and in all genotypes once microalbuminuria appears; not suggested for isolated hematuria in heterozygous individuals. ^g^ Persistent hematuria in all affected males; risk of ESKD depends on genotype. ^h^ Nephrotic syndrome; renal histology not reported. ^i^ *NUP93*, 1–6 y; *NUP107*, 2–3 y. AD, autosomal dominant; AFP, alpha-fetoprotein; AR, autosomal recessive; CAKUT, congenital anomalies of the kidney and urinary tract; CMT, Charcot–Marie–Tooth; CNS, congenital nephrotic syndrome; DMS, diffuse mesangial sclerosis; DSD, disorders of sex development; ESKD, end-stage kidney disease; FSGS, focal segmental glomerulosclerosis; GAMOS, Galloway–Mowat syndrome; HGNC, HUGO Gene Nomenclature Committee; KEOPS, kinase, endopeptidase and other proteins of small size; MCD, minimal change disease; MELAS, mitochondrial encephalomyopathy with lactic acidosis and stroke-like episodes; Mt, mitochondrial; NS, nephrotic syndrome; RAS, renin–angiotensin system; XL, X-linked. Gene symbols follow HGNC nomenclature.

## 7. Convergent Mechanisms of Podocyte Injury, Depletion and Immune-Mediated Damage

**Figure 3:**
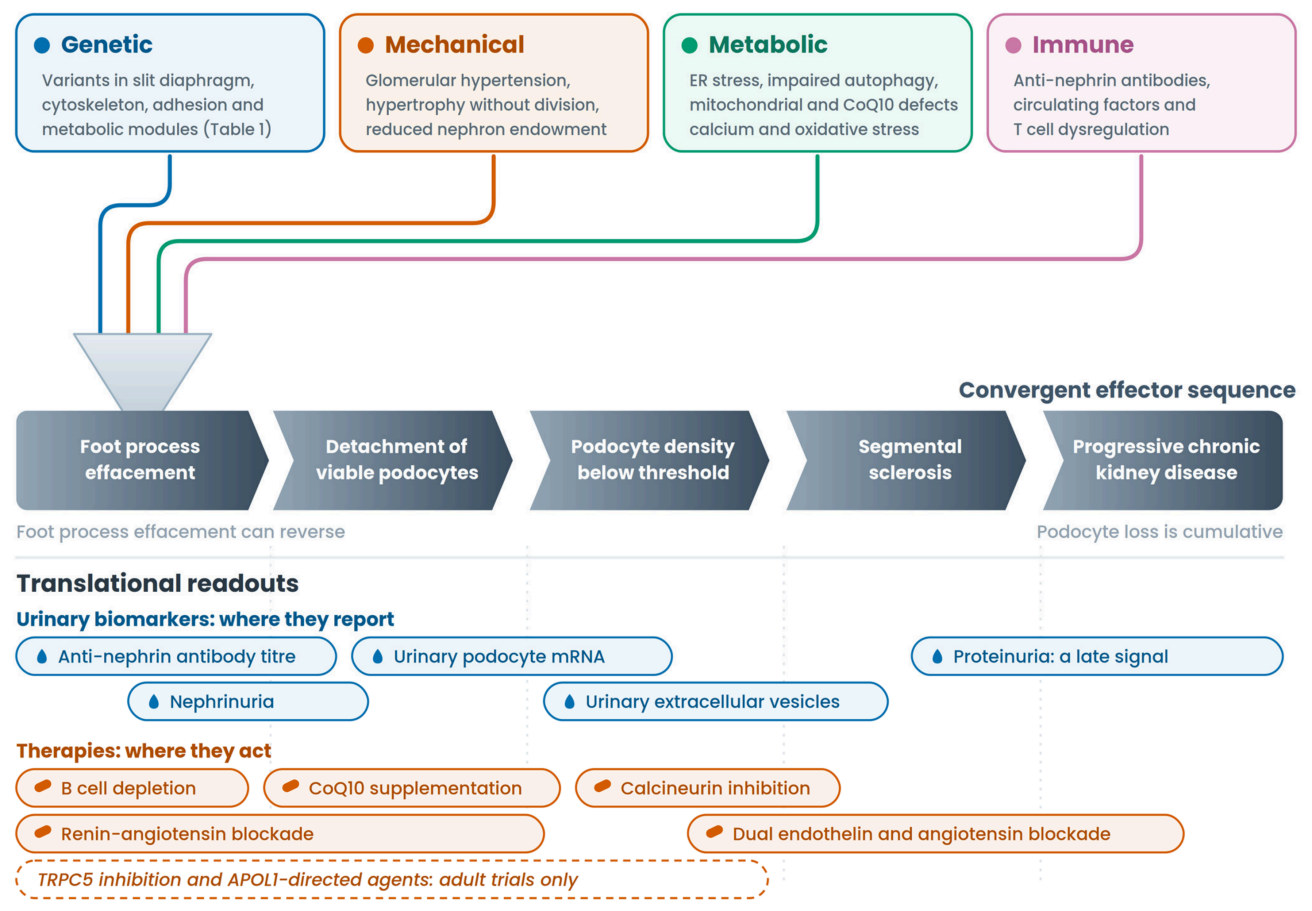
Convergent mechanisms of podocyte injury and their translational readouts. Genetic, mechanical, metabolic and immune insults converge on a common effector sequence of foot process effacement, detachment of viable podocytes, depletion below the density threshold, segmental sclerosis and progressive chronic kidney disease. The lower panel indicates where podocyte-derived urinary biomarkers and serum anti-nephrin antibodies report on this sequence and where current and emerging therapies act. Proteinuria is placed as a late signal of podocyte loss, although it can also arise from reversible effacement alone. Agents drawn with a broken outline have been tested only in adults.

Podocytopathies of very different origin converge on a small number of effector responses, and the glomerulus fails when podocyte density falls below a threshold rather than when any single pathway is activated. Graded podocyte ablation in rats separates that failure into stages: loss of up to 20% produces mesangial expansion and transient proteinuria with preserved function, 21% to 40% adds capsular adhesions and segmental sclerosis with mild persistent proteinuria, and loss beyond 40% gives segmental to global sclerosis, heavy proteinuria and falling filtration [102]. What unifies these stages is not podocyte number but podocyte density, the number of cells per unit of glomerular volume, which is why the same absolute number of podocytes can suffice in a small glomerulus and fail in an enlarged one [103,104]. Every mechanism below acts by moving one of the two terms of that ratio (Figure 3), and each does so through the same two-step sequence: a reversible effacement of foot processes, and then an irreversible loss of cells.

Mechanical loading moves both terms at once. Glomerular capillary hypertension distends the tuft, and because the podocyte can answer an enlarging capillary surface only by hypertrophy, coverage thins as the glomerulus grows. Detachment of viable cells follows from the distending and shear forces acting on a cell that must stay anchored while the surface beneath it expands, and this rather than cell death is the principal route of loss [105]. In children the arithmetic is unforgiving: a reduced nephron endowment raises single-nephron filtration from the outset [5], so the hypertrophic response begins earlier and from a smaller starting population than in an adult meeting the same insult.

A second set of responses concerns protein handling. Mutant nephrin, podocin and laminin beta2 are often retained in the endoplasmic reticulum rather than trafficked, and the resulting load of misfolded protein triggers the unfolded protein response, whose adaptive branches attenuate translation and induce chaperones but whose sustained activation ends in apoptosis [106,107]. This is the basis for the proposal that chemical chaperones might rescue missense alleles that are trafficking defects rather than null alleles, and it is one reason the distinction between truncating and missense variants recurs across every module. Degradation is the other half of proteostasis: podocytes maintain an unusually high basal level of autophagy, and podocyte-specific deletion of Atg5 causes a late-onset glomerulopathy with proteinuria and podocyte loss while raising susceptibility to experimental glomerular injury [108]. Autophagy is suppressed by mTORC1, so the signal that drives hypertrophy also removes the mechanism that clears its cost.

Calcium entry converts mechanical and metabolic signals into cytoskeletal change. TRPC6 and TRPC5 act antagonistically on actin dynamics and podocyte motility, so the phenotype depends on the balance between them rather than on either channel alone [109], and inhibition of TRPC5 protects the filter in experimental injury [110]. Downstream, calcium activates calcineurin, which dephosphorylates synaptopodin and exposes it to degradation, taking with it the contractile architecture of the foot process; blocking that step reduces proteinuria by an effect on the podocyte that does not depend on inhibiting NFAT in T cells [111]. Oxidative and mitochondrial injury feed the same node, most clearly in the coenzyme Q10 pathway.

Epigenetic control is the least developed of these strands, but its best characterized example is instructive. Transgenic expression of miR-193a in mice suppresses WT1 and rapidly induces FSGS, showing that a podocyte can be dedifferentiated by a single regulatory RNA with no coding mutation anywhere [112]. DNA methylation and histone modification are documented in experimental podocyte injury but have not been mapped in pediatric disease.

The fate of the injured cell determines whether loss is recoverable. Apoptosis is readily produced in cultured podocytes but is hard to demonstrate in vivo, whereas podocytes recovered from urine can be cultured, which places detachment of viable cells rather than death at the center of depletion [113]. Cells that remain may dedifferentiate, losing the markers used to count them and therefore exaggerating apparent loss on biopsy. Parietal epithelial cells activate and migrate onto the tuft, contributing to adhesions and to the sclerotic lesion itself rather than to repair, which is the counterpart of the unresolved question of whether these cells can serve as podocyte progenitors [25,114]. This transition, from an effacement that can reverse to a depletion that cannot, is the event that matters prognostically, and it is the event that no measurement currently available in children detects.

Immune injury reaches the same effectors by another route, and its history is a long approach to a defined antigen. The remission of lipoid nephrosis during measles and its response to steroids prompted the proposal in 1974 that the disease is a disorder of T-cell function producing a circulating mediator toxic to the filtration barrier [24]. Candidate factors followed, of which soluble urokinase receptor was the most prominent: it was reported to be raised in two thirds of primary FSGS and to activate podocyte beta3 integrin [115], but reassessment found that recombinant suPAR did not induce proteinuria in wild-type mice and that concentrations in patients tracked filtration rate rather than the diagnosis [18]. The position changed when autoantibodies against nephrin were identified in minimal change disease and in children with idiopathic nephrotic syndrome, and passive transfer of patient antibody reproduced a podocytopathy [12,13,14]. An antibody against a named slit diaphragm protein turns the circulating factor from a hypothesis into a testable mechanism and would account for why disease recurs in a transplanted kidney while genetic forms largely do not, although detection of the antibody at recurrence has depended on the assay used (Section 9.1).

Whether these mechanisms operate in the human pediatric glomerulus, and in what order, depends on which experimental system is used to ask the question.

## 8. Experimental Platforms and Human Tissue Mapping

No single experimental system reproduces the mature human podocyte, and the discrepancies between systems are themselves informative. In adult mouse kidney, single-nucleus sequencing recovered twenty times more podocytes than published single-cell datasets, 2.4% against 0.12% [116], whereas a systematic comparison in the same tissue found the opposite, 0.7% of single-nucleus against 3.28% of single-cell libraries, with warm dissociation recovering three podocytes in total [117]. Both results are correct. The dissociation protocol, rather than the choice between cell and nucleus, sets the result, so any statement about how abundant podocytes are, or which genes they express, is influenced by the sample preparation method.

Spatial methods keep the glomerulus intact but exchange one limit for another. The most widely used barcoded platform captures transcripts in spots of 55 micrometres, so a glomerular spot mixes podocyte, endothelial and mesangial signal that must be assigned computationally to cell types defined elsewhere [118], while imaging-based platforms reach single-cell resolution but read a panel chosen before the experiment.

Organoids derived from induced pluripotent stem cells generate podocytes that express nephrin and podocin and form foot processes, but at a fetal stage. Their cell types benchmark to first and second trimester kidney and off-target proportions vary between healthy donor lines [119]; organoid podocytes express roughly a fifth of the transcription factors of their adult counterparts, and extended culture lowers differentiation markers and adds an off-target muscle population, so the intuitive remedy makes the problem worse [120]. Within those limits the system reproduces disease: organoids from patients with *NPHS1* variants mislocalize nephrin and fail to form a slit diaphragm [121]. A glomerulus chip adds the variable that static culture lacks, cyclic strain across a podocyte and endothelial bilayer, and reconstitutes differential clearance of albumin and inulin and drug-induced albuminuria [122] at the cost of throughput.

Immortalized human podocyte lines remain the workhorse and the largest single source of discordance. They were selected for conditional proliferation and for nephrin and podocin expression [123], but their phenotype is unstable in culture, which is why a glomerulus chip was held to be impossible until functional human podocytes became available [122], and the quasi-crystalline junction has been resolved only in native glomeruli [33,35]. A signaling result obtained in a cell without an assembled slit diaphragm is a hypothesis about the junction rather than an observation of it.

Animal models supply the intact organ. Inducible podocyte-specific deletion in the mouse separates a developmental from a maintenance defect, zebrafish provide a filtering pronephros in a transparent embryo, and *Drosophila* nephrocytes, which carry a slit diaphragm of the same bilayered architecture [34], form a screening platform on which orthologues of human nephrotic syndrome genes reproduce the central pathogenic alterations [124].

Human tissue remains the reference against which all of this is judged and the scarcest material. Cryo-electron tomography of native glomeruli has settled questions that no model could [33,35], but the reference atlases derive from adult tissue [16] and the fetal reference from the first half of gestation [28], so that pediatric values are extrapolated (Section 11). Table 2 compares what each platform resolves and where it has disagreed with another.

The value of these platforms is ultimately measured at the bedside: a way to detect podocyte loss before filtration falls, and a way to choose treatment by mechanism rather than by steroid response.

**Table 2:** Experimental platforms for the study of the podocyte, compared by what they resolve and by where they disagree. Podocyte fidelity refers to how closely the podocyte in that system resembles the mature human cell. Documented discordance gives a published instance in which a platform returned a different answer from another system, with the reason where it is known.

Platform	What It Resolves	Podocyte Fidelity	Throughput	Main Limitations	Documented Discordance	Pediatric Applicability
**Single-nucleus RNA sequencing**	Cell-type composition and podocyte transcriptome	Native cell, nuclear transcripts only	High	Spatial context lost; recovery depends on protocol	Podocyte yield 2.4% vs. 0.12% favoring nuclei [116], but 0.7% vs. 3.28% favoring cells [117]; the direction is set by dissociation, not by cell versus nucleus	Reference atlases adult [16]; fetal data end at week 18 [28]
**Spatial transcriptomics**	Expression kept in glomerular context	Native tissue, podocyte signal mixed with endothelium and mesangium	Moderate	A 55 µm spot exceeds a podocyte; imaging platforms read targeted panels	Cell types assigned by computational deconvolution against a single-cell reference [118], so the assignment inherits its bias	Almost entirely adult nephrectomy and biopsy
**iPSC kidney organoid**	Human podocyte development and monogenic disease	Nephrin and podocin present, foot processes form, maturity fetal	Low to moderate	No perfusion; off-target cells vary between donor lines	Benchmarks to first and second trimester fetal kidney rather than adult [119]; longer culture lowers terminal markers while adding off-target cells [120]	Strongest here: patient NPHS1 lines mislocalize nephrin [121]
**Glomerulus on a chip**	Filtration and mechanical loading	Mature markers and foot processes, no three-dimensional tuft	Low	Few questions at scale; artificial membrane in most designs	Reconstitutes differential clearance of albumin and inulin [122], which cell lines do not; built because functional human podocytes were unavailable in culture	Patient-derived chips demonstrated; no pediatric cohort
**Immortalized podocyte line**	Signaling and cytoskeletal responses in one cell	Lowest, phenotype unstable and junction not assembled	Highest	No slit diaphragm; no mechanical or matrix context	Selected for nephrin and podocin expression [123], yet the assembled fishnet junction has been resolved only in native tissue [33,35]	Not age-specific; patient-derived lines available
**Animal models**	Gene function in an intact, perfused, growing organ	Native podocyte, non-human	Mouse low, zebrafish and fly high	Species differences; nephrogenesis timing differs from human	Human and mouse renal embryogenesis differ pervasively [28], yet Drosophila nephrocytes reproduce the central alterations of human nephrotic syndrome genes [124]	Direct: models exist for most genes in Table 1
**Human biopsy**	Diagnosis, in situ architecture and fine structure	Highest; the reference against which the rest is judged	Very low	Tissue scarce; fixation alters what is seen	The zipper model from chemically fixed tissue [23] was revised to a flexible scaffold [32] and then to a fishnet by cryo-electron tomography [33,35]	Pediatric biopsies are few, small and taken for clinical indications

iPSC, induced pluripotent stem cell. Throughput is given relative to the other platforms in this table rather than in absolute terms.

## 9. Podocyte-Derived Urinary Biomarkers and Genotype-Guided Therapy

### 9.1. Biomarkers of Podocyte Injury and Loss

Proteinuria is an early signal of podocyte injury but an indirect and late signal of podocyte loss, and the reason is structural rather than analytical. Foot process effacement is a reorganization of the actin cytoskeleton that can reverse, whereas the detachment of viable podocytes is cumulative and cannot; the two are distinct events separated in Section 5 and in Figure 3, and proteinuria reports directly on the first and only indirectly on the second. Proteinuria, even in the nephrotic range, can arise from effacement and remit completely, as it does in steroid-sensitive nephrotic syndrome [2], so its presence does not by itself indicate that podocytes have been lost. Conversely, in rats with graded experimental depletion, the loss of up to a fifth of podocytes was accompanied only by transient proteinuria [102]. Proteinuria alone therefore establishes neither whether irreversible podocyte loss has occurred nor how extensive it is. The markers considered here are attempts to detect that loss without a biopsy, and they do not all report on the same point of the sequence. Table 3 classifies each by the population in which it was studied, by study design and by validation status.

Podocyte mRNA in the urinary pellet is the most developed attempt to count cells rather than to measure leak. The method was established in a rat model of graded podocyte depletion, where nephrin and podocin transcripts in urine particulates reported cell loss from the glomerulus [125], and it was then applied to patients: across 358 adult and pediatric clinic attenders and 291 controls, urine podocyte transcripts were 79-fold above control in patients whose biopsy-proven glomerular disease had progressed, rose with disease activity and returned towards baseline in remission [126]. Two features of that study set the limit of what can be claimed from it. Children and adults were pooled and no pediatric subgroup was analyzed, and no multivariable model was fitted, so the marker was shown to accompany progression but not to add information beyond proteinuria and filtration rate. Urine nephrin mRNA also fell with age across the cohort [126], which is the empirical form of the problem set out in Section 11: podocyte density falls with normal growth, so a pediatric threshold cannot be borrowed from adults.

Direct counting of urinary podocytes and measurement of urinary nephrin extend the same idea to whole cells and to protein. Podocalyxin-positive cells are absent from the urine of healthy children and of children with non-inflammatory glomerular disease and are detectable in inflammatory glomerular disease in proportion to activity [127]; in a series of 20 patients aged 4 to 24 years followed across serial biopsies, it was cumulative rather than single-point podocyturia that tracked chronic lesions and glomerulosclerosis [128]. Urinary nephrin measured by enzyme-linked immunosorbent assay was detectable in about half of 65 treatment-naive children with glomerular disease and correlated with the albumin-to-creatinine ratio and with urinary podocyte count [129]. These studies are cross-sectional or small, and none has an agreed denominator: results are variously normalized to creatinine, to urine volume or to a counted field.

Urinary extracellular vesicles carry the same signal in another compartment, and the pediatric evidence here is easily over-read. Higher urinary exosomal miR-193a distinguished primary FSGS from minimal change nephropathy and IgA nephropathy in children, with areas under the receiver operating characteristic curve of 0.85 and 0.82, respectively, and correlated with the glomerulosclerosis index on biopsy [130]. The study comprised 25 children in total, of whom eight had primary FSGS; the authors describe it as cross-sectional, and no clinical outcome was observed. The reported association with prognosis is therefore an association with a histological score rather than with kidney survival, and the study is a single-center discovery series without a validation cohort.

Two further candidates show how quickly this field can generate a biomarker and then lose it. Urinary CD80 rises in children with idiopathic minimal change disease in relapse and separates relapse from remission, whereas serum CD80 does not [131], and podocyte B7-1 staining was used to select five patients for abatacept, all of whom achieved partial or complete remission of proteinuria [132]; independent groups were then unable to reproduce the staining, and the value of B7-1 as a biomarker is disputed [19]. Soluble urokinase receptor followed the same trajectory for different reasons, with the reassessment described in Section 7 having found that concentrations track filtration rate rather than the diagnosis [18,115]. Neither is in routine pediatric use.

Antibodies against nephrin are the only marker in this group that reports on a defined mechanism rather than on accumulated damage, and they are also the marker whose status is changing fastest. Autoantibodies were identified in minimal change disease [12] and then measured across a multicenter series of 357 adults and 182 children, where they were present in 44% of adult minimal change disease and 52% of children with idiopathic nephrotic syndrome, rising to 69% and 90% respectively in immunosuppression-naive patients with active disease, and were rare in membranous nephropathy and in controls, with titres correlating with disease activity at inclusion and during follow-up [13]. In 333 children the prevalence separates immune from non-immune disease: 68% in steroid-sensitive and 28% in steroid-dependent nephrotic syndrome against 14% in non-genetic and 2% in genetic steroid-resistant disease. Among children with non-genetic steroid-resistant disease and active nephrosis, seropositivity occurred in 18% of those who responded to intensified immunosuppression and in none of those with multidrug resistance [15]. Passive transfer of patient antibody reproduces a podocytopathy with minimal change lesions in mice [14], which is what distinguishes this marker from every preceding candidate circulating factor.

The measurement, however, is not yet a test, and this qualification has become more rather than less important since the original reports. Every prevalence figure above was generated by in-house immunoprecipitation or immunoprecipitation-ELISA using human nephrin ectodomain expressed in human cells, and the result depends on the antigen and the platform. A head-to-head comparison of conventional ELISA, bead-based ELISA, immunoprecipitation–Western blot and tissue immunofluorescence across 169 participants found that antigen produced in murine cells generated false-positive signals, including in samples from healthy controls, and that a human-cell antigen combined with an immunoprecipitation-based assay gave the most robust results [20,133]. The Immunonephrology Working Group of the European Renal Association notes that reported positivity rates span approximately 19% to 91% according to assay and population and that no standardized or validated commercial assay is available [21]; the groups who developed the assays have set out the same pitfalls, including commercial preparations whose antigen covers only a small fraction of the human nephrin ectodomain and false positives following recent infection [133]. Most consequentially, an independent study of 65 patients with steroid-sensitive or steroid-resistant nephrotic syndrome, most of them adults, together with 34 patients with post-transplant recurrence and 20 healthy volunteers, found that positivity obtained with a murine-cell antigen was highest in the healthy volunteers, at 55%, and tracked naturally occurring anti-alpha-galactosyl antibodies, while with human-cell nephrin positivity fell below 5% across the patient groups and was comparable to that in healthy volunteers [22]. A KDIGO controversies conference summarized the position as low circulating titres with detection techniques that have proved technically challenging and difficult to reproduce and made no recommendation for routine testing [134]. Anti-nephrin autoantibodies are therefore a mechanistically important finding and a research assay and not yet a pediatric diagnostic test.

Three limitations apply to every candidate marker in Table 3, that is, to every entry other than the proteinuria comparator, and are stated here rather than distributed through it. No analyte in this group has an agreed reference material, an agreed denominator or published analytic performance across laboratories, so a value obtained in one center cannot be set beside a value obtained in another. No pediatric reference interval or decision threshold has been established for any of them; the threshold most widely used in the anti-nephrin literature was set at the highest healthy-control titre in a single cohort [12]. And the evidence is predominantly cross-sectional: the diagnostic discrimination reported for urinary vesicle miR-193a comes from a single series without a validation cohort [130], and no study in children has shown that a podocyte-derived urinary marker adds prognostic information beyond proteinuria and estimated filtration rate in an adjusted model. The nearest pediatric attempt, a 24-month prospective study of the urinary podocalyxin-to-creatinine ratio in 169 children with chronic kidney disease, adjusted for age and primary diagnosis but not for baseline proteinuria or filtration rate, and its authors concluded that the marker was not useful in stages 1 to 3 [135]. The one pediatric cohort study that has shown improved discrimination of progression with a biomarker panel, raising the C statistic from 0.76 to 0.85 in 599 children, contains no podocyte-derived analyte [136]. Until that comparison is made, the claim available for this group of markers is that they report disease activity and cell loss, not that they should change a decision.

**Table 3:** Podocyte-derived and mechanism-directed markers by population, study design, validation status and current clinical applicability. Population class follows Section 2.3 (P, pediatric human; M, mixed pediatric and adult; X, preclinical). Established denotes support from a clinical practice recommendation or reproducible pediatric cohort data; emerging denotes human data that include children, from multicenter or longitudinal studies, which are not yet standardized or prospectively validated; experimental denotes preclinical or early-phase adult data or a single cross-sectional discovery series; not supported denotes an original finding not confirmed on reassessment or no added value in a prospective study. Proteinuria is included as the comparator.

Marker	Population (Class)	Study Design	Validation Status	Current Clinical Applicability	Ref.
Urinary podocyte mRNA	Rat (X); 358 adults and children, 291 controls (M)	Animal model; clinic-based cross-sectional study with serial sampling	Emerging	Research use only; no pediatric reference values; not compared with proteinuria in an adjusted model	[125,126]
Urinary podocyte count	Mainly children (P)	Cross-sectional; longitudinal series of 20 patients	Emerging	Research use only; no standardized counting method	[127,128]
Urinary nephrin	65 children (P)	Cross-sectional, single center	Experimental	Research use only	[129]
Urinary podocalyxin-to-creatinine ratio	169 children with CKD (P)	Prospective cohort, 24 months; not adjusted for proteinuria	Not supported	No added value in CKD stages 1 to 3	[135]
Urinary vesicle miR-193a	25 children (P)	Cross-sectional, single center; no clinical outcome	Experimental	Research use only; no validation cohort	[130]
Urinary CD80	Children with MCD (P); B7-1 staining studies (M)	Cross-sectional group comparison	Experimental	Not in use; the related podocyte B7-1 staining was not reproduced	[19,131,132]
suPAR	Adults and children (M)	Case–control series; reassessment in a prospective cohort	Not supported	Not in use; levels track filtration rate	[18,115]
Anti-nephrin antibodies	357 adults and 182 children (M); 333 children (P); mice (X)	Multicenter cross-sectional serology with follow-up; passive transfer in mice	Emerging	Research use only; no standardized assay; positivity varies with assay and population	[12,13,14,15,20,21,22,133]
Proteinuria (comparator)	Children (P)	Guideline; prospective cohort	Established	Routine; the reference against which new markers must add value	[8,136]

CKD, chronic kidney disease; MCD, minimal change disease; suPAR, soluble urokinase plasminogen activator receptor. Apart from proteinuria, no marker in this table has an agreed reference material, published inter-laboratory analytic performance or a pediatric reference interval.

### 9.2. Genotype-Guided and Mechanism-Directed Treatment

Genotype-guided and mechanism-directed treatment in children currently comprises a small set of established consequences, two emerging approaches, one serological and one mechanism-directed, and a group of experimental approaches that have only been tested, if at all, in adults. The distinction matters because these are frequently discussed together, and the groups are separated here and again in Table 4.

Of the established consequences, the first is subtractive and the most widely applicable. In the PodoNet cohort a confirmed genetic diagnosis, and not the histological pattern, predicted the response to intensified immunosuppression [54], and current recommendations accordingly advise withholding calcineurin inhibition and stopping prednisolone in children with evidence of a monogenic form [8], a treatment whose toxicity in a growing child is considerable. The rule is not absolute, and the exception is worth quantifying: in a systematic review of 178 genetic cases, 35% responded fully or partially to calcineurin inhibition, most often where the biopsy showed minimal change [137]. A genetic result therefore argues against prolonged immunosuppression rather than against any trial of it, and where treatment is withdrawn the guideline requires that the variant first be reviewed for pathogenicity and that the decision be taken with the family [8].

The second is the clearest positive consequence and is confined to one pathway. Among 116 patients supplemented with coenzyme Q10 after a genetic diagnosis, proteinuria fell by 88% at twelve months; in 41 treated children with chronic kidney disease stages 1 to 4, five-year kidney failure-free survival was 62% against 19% in untreated controls matched for genotype, age, kidney function and proteinuria [94]. The exception carries the argument: no nephroprotective effect was seen in *COQ8B* disease, which the authors attribute to its later diagnosis and more advanced stage at the start of treatment, so the benefit belongs to early treatment rather than to the drug alone.

The third and fourth alter surveillance and timing rather than drug choice. A *WT1* genotype carries a risk of Wilms tumor and, in dysgenetic gonads, of gonadoblastoma, so it triggers tumor imaging, karyotyping and a decision about gonadectomy, and it makes native nephrectomy before transplantation a question that has to be asked [59,60]. In the collagen IV disorders the intervention is early renin–angiotensin blockade, and the guideline now specifies the timing by genotype and sex: treatment is suggested in males with X-linked disease and in both sexes with autosomal recessive disease from two years of age once microscopic hematuria is present, and is titrated to the maximum tolerated dose in all genotypes once microalbuminuria appears, whereas it is not suggested in heterozygous individuals with isolated microscopic hematuria [9]. The pediatric randomized trial of early treatment was under-enrolled and its result should be reported as such: among randomized children, three of 11 progressed on ramipril against five of nine on placebo, an almost halved risk that did not reach conventional significance, with adverse events no more frequent than on placebo [138]. The recommendation rests on that trial together with registry data [9].

The fifth concerns transplantation, where the genotype changes the estimate of risk on both sides of the operation. The difference in recurrence risk between genetic and non-genetic disease noted in Section 6 [54] is reproduced in a national cohort, with no recurrence in genetic disease against a 47.8% recurrence risk in primary steroid resistance without an identified variant [139]. On the donor side, the Alport guideline recommends determining the genotype in all possible related donors and advises against donation by a heterozygous carrier under 40 years of age [9]. Congenital nephrotic syndrome is the instructive exception to the rule that genetic disease does not recur: children homozygous for the *NPHS1* truncating Fin-major variant have never expressed nephrin, and de novo post-transplant glomerulopathy occurs in 25% to 35% of them, where at least 70% of whom have detectable anti-nephrin antibodies raised by alloimmunization against the graft [6].

Two developments are emerging rather than established. The first is the use of an antibody titre to guide B cell depletion rather than treating by relapse, which follows logically from the observation that titres track activity [13,15] but has not been tested prospectively in children and depends on an assay that is not yet standardized (Section 9.1). The second is mechanism-directed rather than genotype-guided. Dual endothelin and angiotensin receptor blockade has the only meaningful pediatric exposure among the newer agents: the phase 3 trial in FSGS enrolled from eight years of age at its United States and United Kingdom sites, 35 of 371 participants were children, and treatment improved partial remission of proteinuria at the week 36 interim analysis but produced no significant difference in filtration slope at week 108 [140].

The remainder is experimental and should be presented to families as such. Inhibition of TRPC5 protects the filter in experimental injury [110], and a selective inhibitor has entered a phase 2a trial in adults [141]. The APOL1 channel inhibitor inaxaplin reduced the urinary protein to creatinine ratio by 48% at thirteen weeks in adherent participants of a phase 2a study in sixteen adults with two *APOL1* variants and biopsy-proven FSGS [142]. Chemical chaperones for missense alleles that are trafficking defects (Section 7) remain preclinical [106,107]. None of these has been tested in children.

Taken together, genotype-guided therapy in pediatric nephrology at present means, in descending order of certainty, the withdrawal of a treatment unlikely to work, a vitamin-like supplement in one biosynthetic pathway, a surveillance program in one gene, the timing of a drug class in the collagen IV disorders, and a revised estimate of transplant risk. It does not yet mean choosing a drug because of a variant, outside the coenzyme Q10 pathway. Describing it in those terms is not a diminution of the field but a statement of where its boundary currently lies.

## 10. Integrating Histopathology, Genetics and Biomarkers

### 10.1. The Continuing Role of Kidney Biopsy

The account given in Section 6 and Section 9 could be read as describing the replacement of histopathology by molecular diagnosis. It is not, and the guidelines are explicit about where each has primacy. The kidney biopsy remains the reference standard for the diagnostic evaluation of glomerular disease [143], and in children it does three things that no molecular test does: it identifies the secondary glomerulopathies in which podocyte injury is downstream of a systemic process, it establishes the pattern and the chronicity of injury, and it supplies the tissue against which any future measure of podocyte density must be calibrated.

Where genetic testing has displaced biopsy, it has done so for defined phenotypes rather than in general. In congenital nephrotic syndrome, comprehensive genetic screening of the podocytopathy genes is recommended as the first-line diagnostic measure in every patient, routine biopsy is not recommended, and biopsy is reserved for sporadic non-syndromic disease in which testing has not produced a molecular diagnosis, or for a substantial fall in filtration rate in which a rare alternative diagnosis is being sought [6]. In Alport syndrome, biopsy is not needed once a genetic diagnosis of X-linked or autosomal recessive disease has been made, but retains a role in heterozygous *COL4A3* or *COL4A4* carriers, in whom a second glomerular disease may coexist, and in the evaluation of an older potential living donor [9]. In SRNS the two are complementary rather than sequential: biopsy is recommended in all children with the diagnosis except in infection- or malignancy-associated secondary disease and potentially in familial, syndromic or already genetically defined cases, while genetic testing is recommended in all children with primary disease and is suggested before biopsy where the result will be available within a few weeks [8]. In steroid-sensitive disease neither is recommended at presentation in a child older than one year with typical features; biopsy becomes indicated with atypical features such as macroscopic hematuria, low C3, sustained hypertension or acute kidney injury not related to hypovolemia, and both are considered in infantile onset between three and twelve months, where about half of cases prove to be genetic [2]. A more recent guideline reaches the same position from the other direction, stating that because prognosis is best predicted by the response to initial treatment and by relapse frequency, a biopsy is not usually needed at presentation and is reserved for resistance to therapy or an atypical course [144].

What happens when genetic testing is negative or inconclusive is the situation that a molecular framing handles least well, and it is also the commonest. The yield of panel testing in SRNS beyond the first year of life is of the order of 10% to 25% [53,54,56], so most children tested will have no causative variant, and a negative result is not evidence of an immune mechanism. Three consequences follow. First, histology carries the diagnosis: the distinction between minimal change and a segmental sclerosing lesion, together with the extent of chronic damage, remains the best available guide to treatment and prognosis, and in a national pediatric cohort the biopsy pattern did not correlate systematically with the genetic result, which is precisely why the two cannot substitute for one another [139]. Second, a negative panel has a differential of its own: a phenocopy gene outside the panel, a structural or deep intronic variant not covered by the assay, a variant classified as uncertain, or genuinely acquired disease. Extended sequencing with reverse phenotyping resolves a substantial proportion of such cases (Section 6) [68], and periodic reanalysis of unsolved cases as classification evidence accumulates is explicitly recommended [74]. Third, the absence of a variant is what makes the immunological question worth asking at all; it is in this group, and not in genetic disease, that anti-nephrin serology would find its use if it becomes measurable to a clinical standard (Section 9.1).

### 10.2. Situations in Which a Molecular Result Changes Management

Table 4 states, for each situation in which a molecular or serological result changes a clinical decision in a child, what is tested, what follows from the result, the design of the supporting studies and how far the evidence extends. Three features of that table are worth drawing out. The consequences that are established are concentrated at the beginning of the illness and around transplantation and are largely decisions about what not to do or when to start something already available, rather than about which drug to choose. Coenzyme Q10 supplementation is the single established instance of a drug chosen because of a variant; after transplantation in *NPHS1*-associated disease, the genotype prompts the search for antibodies, and it is the antibody that directs treatment. And every entry classified as emerging or experimental depends either on an assay that is not standardized or on evidence obtained only in adults.

**Table 4:** Situations in which a molecular or serological result changes, or may come to change, clinical management in a child. The validation status (established, emerging or experimental) follows Section 2.3, with the population class of the supporting evidence in parentheses (P, pediatric human; A, adult human; M, mixed).

Clinical Situation	Test and Result	Consequence for Management	Study Design	Validation Status (Class)	Ref.
Primary SRNS	Causative podocytopathy variant	Withhold or withdraw calcineurin inhibition and stop glucocorticoids after reviewing the variant; continue RAS blockade. About a third of genetic cases respond at least partially.	Guideline; registry cohort; systematic review	Established (P)	[8,54,137]
SRNS, coenzyme Q10 pathway	*COQ2*, *COQ6*, *COQ8B* or *PDSS2* variant	Start CoQ10 early; the only established drug chosen because of a variant	Guideline; registry cohort with matched controls	Established (P)	[8,63,94]
Any presentation	Pathogenic *WT1* variant	Wilms tumor imaging, karyotype and gonadectomy decision; consider native nephrectomy before transplantation	Registry and national cohorts	Established (P, M)	[59,60]
Persistent glomerular hematuria	*COL4A3*, *COL4A4* or *COL4A5* variant	RAS blockade from 2 years once hematuria is present in X-linked males and autosomal recessive disease; in all genotypes once microalbuminuria appears	Guideline; small randomized trial	Established (P, M)	[9,138]
Before transplantation	Variant found or not found	Recurrence 4.5% with and 25.8% without a genetic diagnosis; genotype all related donors	Registry and national cohorts; guideline	Established (P, M)	[9,54,139]
CNS with post-transplant proteinuria	Homozygous *NPHS1* Fin-major variant	Consider anti-nephrin-mediated disease and antibody-directed treatment	Consensus recommendation	Established (P)	[6]
SRNS without a variant	Negative gene panel	Histology guides treatment; reverse phenotyping, reanalyze, and consider an immune cause	Guideline; cohort studies; consensus conference	Established (P, M)	[8,68,74,139]
Steroid-sensitive or steroid-dependent NS	Anti-nephrin antibody titre	Could guide B cell depletion; not tested prospectively and not standardized	Multicenter serology with follow-up	Emerging (P, M)	[13,15,21,134]
FSGS with two *APOL1* risk variants	*APOL1* high-risk genotype	APOL1 channel inhibition lowered proteinuria in 16 adults; no pediatric data	Pediatric cohorts; phase 2a trial	Experimental (A, P)	[71,72,142]

CNS, congenital nephrotic syndrome; CoQ10, coenzyme Q10; FSGS, focal segmental glomerulosclerosis; NS, nephrotic syndrome; RAS, renin–angiotensin system; SRNS, steroid-resistant nephrotic syndrome.

### 10.3. A Practical Diagnostic Sequence

**Figure 4:**
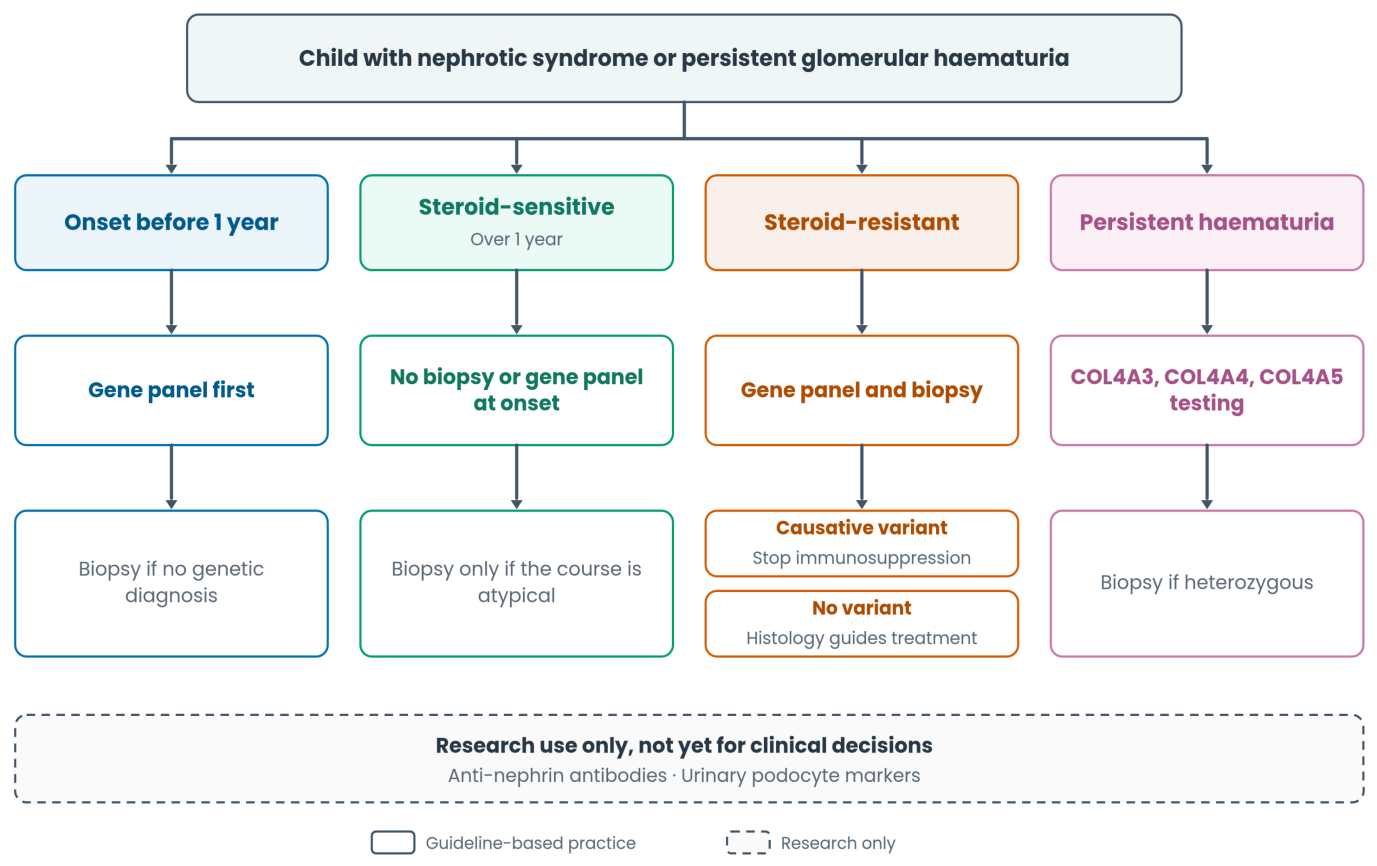
Integrated diagnostic sequence in pediatric glomerular disease, entered by age at presentation, response to glucocorticoid and the presence of persistent glomerular hematuria. Solid outlines indicate practice supported by a guideline recommendation; the broken outline indicates measurements not yet standardized or validated for clinical use in children. The figure is a synthesis of the clinical practice recommendations cited in Section 10.1, not a new or validated algorithm.

Figure 4 sets out how these elements combine in practice. For nephrotic syndrome the sequence is organized by the two variables that carry the most information in a child, the age at presentation and the response to an adequate course of glucocorticoid, because these determine both the prior probability of a monogenic cause and the urgency of the decisions that follow; persistent glomerular hematuria is a separate point of entry. Presentation before one year leads to genetic testing first, with biopsy held in reserve for children without a molecular diagnosis, although between three and twelve months of age both may be considered at once; typical steroid-sensitive disease after one year requires neither at presentation; steroid resistance triggers both, with the order set by how quickly the genetic result can be obtained; and persistent glomerular hematuria leads to targeted collagen IV testing, with biopsy retained mainly for heterozygous carriers. The markers discussed in Section 9.1 are shown separately, with a broken outline, because none is yet measurable to a clinical standard. The figure is a synthesis of the recommendations cited in Section 10.1 rather than a new or validated algorithm, and it is offered as a framework for locating the biology reviewed here within current practice, not as a substitute for the guidelines from which it is drawn.

## 11. Limitations

Several limitations qualify this review. They fall into two groups: those of the evidence it summarizes, and those of the method by which it was assembled.

The evidence base is constrained first by the scarcity of pediatric human tissue. Biopsies from children are obtained only for clinical indications and leave little material for research, the atlases used as the reference for glomerular cell states derive from adult nephrectomy and deceased donor kidneys [16], and the fetal reference ends at 18 weeks of gestation [28]. Between these two references, pediatric values for nearly every quantity discussed here are extrapolated rather than measured (Section 8).

Second, no pediatric reference values exist for any of the markers considered in Section 9.1, and the obstacle is physiological rather than administrative. Podocyte number is fixed before birth while glomerular volume increases throughout childhood, so podocyte density falls with normal growth and an adult-derived threshold describes a different physiology when applied to a young child. The single age effect that has been measured, a fall in urinary nephrin mRNA with age [126], indicates the size of the problem rather than solving it.

Third, much of the mechanistic account rests on preclinical and adult human work, and the classification in Table S1 makes the extent of this explicit rather than removing it. The structural modules described in Section 4 and Section 5 are defined largely by murine genetics and by structural biology performed in murine or adult human tissue; the slit diaphragm architecture on which the account of mechanotransduction depends has been resolved in high-pressure frozen murine glomeruli and in native human tissue from a single adult nephrectomy [33,35], not in pediatric material. The experimental platforms compared in Section 8 each depart from the mature human podocyte in a defined direction, and their documented disagreements are set out in Table 2; organoid podocytes benchmark to first and second trimester fetal kidney [119], with the consequence that the dominant disorders presenting in adolescence have no faithful human model at the age at which they present. Findings from these systems are classified X in Table S1 and are not presented as established in children, but classification does not remove the asymmetry.

Fourth, the biomarker literature is predominantly cross-sectional and small. The largest study of urinary podocyte transcripts pooled children with adults, fitted no multivariable model and analyzed no pediatric subgroup [126]; the pediatric extracellular vesicle study comprised 25 children and observed no clinical outcome [130]; and no study in children has tested a podocyte-derived marker against kidney survival with adjustment for proteinuria and filtration rate. Where a marker has been reported as prognostic, the endpoint has usually been a histological score or a treatment response rather than an observed clinical outcome. For anti-nephrin antibodies the limitation is analytical rather than only observational: prevalence estimates differ several-fold between assays and laboratories, from about half of children with idiopathic nephrotic syndrome in studies using immunoprecipitation-based assays to below 5% across patient groups in an independent series [13,20,21,22]. The figures quoted in Section 9.1 should be read with that dispute in view.

Fifth, several therapeutic results discussed in Section 9.2 derive from adults or from trials in which children were a small minority: 35 of 371 participants in the phase 3 trial of dual endothelin and angiotensin receptor blockade in FSGS were children [140], TRPC5 inhibition has been studied in experimental models and in an adult phase 2a trial [110,141], and APOL1 channel inhibition only in adults [142]. The pediatric trial of early renin–angiotensin blockade in Alport syndrome was under-enrolled and its principal comparison did not reach conventional significance [138].

The method has its own limitations. This is a narrative and not a systematic review: the search was structured but not registered, selection was purposive, no formal risk-of-bias instrument was applied and no quantitative synthesis was attempted, so the review cannot exclude the possibility that a differently conducted search would weight the evidence differently. Screening was performed by one author with review by two others, so selection bias towards the topics and groups most familiar to the authors cannot be excluded, and the restriction to English-language publications will have excluded relevant work, including from some of the populations in which diagnostic yield is highest. The four-way classification of the evidence used in Table S1 is a pragmatic device rather than a validated instrument: assigning a single population to a review, a guideline or a study combining human and animal work requires judgement, and readers may reasonably classify individual sources differently. Finally, the field is moving quickly, and two of the areas covered here, the architecture of the slit diaphragm and the status of anti-nephrin autoantibodies, changed materially during the preparation of this review; the account given is current to the search dates stated in Section 2.1.

## 12. Conclusions and Research Priorities

Two statements can be made about this field with confidence, and they should not be conflated. The first concerns present practice. In a child with nephrotic syndrome, management still rests on the age at presentation, the response to glucocorticoid, histopathology where it is indicated, and genetic testing where it is available; and within that framework a molecular result already changes decisions in a defined and limited set of situations, principally the withdrawal of immunosuppression unlikely to be effective, supplementation in coenzyme Q10 deficiency, tumor and gonadal surveillance in *WT1* disease, the timing of renin–angiotensin blockade in the collagen IV disorders, and the estimation of recurrence risk and evaluation of living donors before transplantation (Table 4, Figure 4). None of these displaces the conventional diagnostic pathway. Each is an addition to it, and the kidney biopsy remains its reference standard.

The second concerns what is not yet available. Nothing in current practice tells a clinician how many podocytes a given child has lost, or how quickly, and no podocyte-derived urinary marker has been shown in children to add information beyond proteinuria and filtration rate. Anti-nephrin autoantibodies are among the most important mechanistic advances in this field and are not yet a clinical test. Genotype-guided treatment in the strict sense, meaning a drug chosen because of a variant, exists for one pathway. That gap, rather than any missing mechanism, is what separates the biology reviewed here from its use at the bedside. Four priorities follow, in the order in which each blocks the others.

The first is standardization. Urinary podocyte transcripts, nephrinuria and anti-nephrin titres are each measured by assays that differ between laboratories in antigen, extraction, normalization and reporting, so results from different centers are not interchangeable; the anti-nephrin literature, in which reported positivity ranges from 19% to 91% according to assay and population and in which the choice of expression system determines whether healthy volunteers appear seropositive, is the clearest demonstration that this is not a secondary concern [21,22]. A marker that cannot be compared between laboratories cannot support a treatment decision, and no amount of further discovery will substitute for reference materials, an agreed denominator and published analytic performance.

The second is pediatric reference values. For the physiological reason set out in Section 11, an adult-derived threshold cannot simply be transferred to a child, and what is required is age-, sex- and body-size-specific ranges for every marker that is proposed, derived from children who are not ill, which is precisely the population from whom tissue and repeated samples are hardest to obtain.

The third is longitudinal cohorts in which genotype and marker are measured in the same child over time. Almost everything published so far is cross-sectional, so a raised value can be shown to accompany disease but not to precede it, and no pediatric study has yet tested a podocyte-derived marker against proteinuria and filtration rate in an adjusted model. A prospective pediatric cohort sampled at fixed intervals from presentation, with sequencing, antibody titre and urinary podocyte markers on the same specimens, would allow a marker to be tested against kidney survival rather than against proteinuria, which can reflect reversible effacement and cannot by itself establish whether, or how many, podocytes have been lost. The design already exists in pediatric nephrology: a urinary and plasma panel improved the discrimination of chronic kidney disease progression in 599 children, and what it lacked was a podocyte-derived analyte [136].

The fourth is a human model that reaches maturity. The limits of organoids and immortalized lines are set out in Table 2 and Section 11. Transplantation, which reduces off-target cells in organoids [119], and sustained flow, which promotes their vascularization and maturation [145], are the two interventions that have moved the phenotype furthest so far, and both deserve to be pushed harder.

These are questions of cohorts, standards and infrastructure, and they become answerable if the networks that built the genetic registries turn to the same task for markers of podocyte loss. The case for detecting that loss early is strongest in exactly the group for whom the evidence is thinnest: the child, whose endowment was fixed before birth and whose whole trajectory is still ahead.

## Data Availability

No new data were created or analyzed in this study. Data sharing is not applicable to this article.

## Supplementary Materials

The following supporting information can be downloaded at https://www.mdpi.com/article/10.3390/life16101722/s1. Table S1: Classification of all cited references by the population in which the primary evidence was generated (P, pediatric human; A, adult human; M, mixed pediatric and adult; X, preclinical).Classification of all 145 cited references by the population in which the primary evidence was generated. Reference numbers correspond to the reference list of the main article. The classification scheme is described in Section 2.3 and its limitations in Section 11.

## Abbreviations

The following abbreviations are used in this manuscript:

AD	autosomal dominant
AFP	alpha-fetoprotein
AR	autosomal recessive
CAKUT	congenital anomalies of the kidney and urinary tract
CMT	Charcot–Marie–Tooth disease
CNS	congenital nephrotic syndrome
CKD	chronic kidney disease
CoQ10	coenzyme Q10
DMS	diffuse mesangial sclerosis
DSD	disorders of sex development
ELISA	enzyme-linked immunosorbent assay
ERA	European Renal Association
ERKNet	European Rare Kidney Disease Reference Network
ESKD	end-stage kidney disease
ESPN	European Society for Paediatric Nephrology
FSGS	focal segmental glomerulosclerosis
GAMOS	Galloway–Mowat syndrome
GBM	glomerular basement membrane
HGNC	HUGO Gene Nomenclature Committee
IPNA	International Pediatric Nephrology Association
iPSC	induced pluripotent stem cell
KEOPS	kinase, endopeptidase and other proteins of small size
KDIGO	Kidney Disease: Improving Global Outcomes
MCD	minimal change disease
MELAS	mitochondrial encephalomyopathy with lactic acidosis and stroke-like episodes
Mt	mitochondrial inheritance
NS	nephrotic syndrome
PRISMA	Preferred Reporting Items for Systematic Reviews and Meta-Analyses
RAS	renin–angiotensin system
SANRA	Scale for the Assessment of Narrative Review Articles
SRNS	steroid-resistant nephrotic syndrome
suPAR	soluble urokinase plasminogen activator receptor
XL	X-linked
