# Supplementary material for: Podocyte Biology in Pediatric Glomerular Disease: From Slit Diaphragm Assembly to Genotype-Guided Therapy—A Narrative Review
Source: Life (Basel). 2026 Oct 15;16(10):1722. doi: 10.3390/life16101722 (PMC13706450; doi:10.3390/life16101722)
Supplement: Supplementary file 1 [file life-16-01722-s001.zip › life-4600621-supplementary.pdf]

Supplementary Materials

Table S1. Classification of all 145 cited references by the population in which the primary evidence was generated. Reference numbers correspond to the reference list of the main article. The classification scheme is described in Section 2.3 and its limitations in Section 11.

Class	Population in which the primary evidence was generated	*n*	Reference numbers
**P**	Pediatric human, including human fetal and neonatal material	57	1–3, 5–8, 10, 11, 15, 26–28, 48, 53–59, 61–65, 68, 69, 71–73, 77–79, 82, 83, 85–88, 94–99, 127–131, 135–139, 144
**A**	Adult human	12	16, 35, 46, 47, 49, 76, 80, 92, 100, 118, 141, 142
**M**	Mixed pediatric and adult cohorts; reviews, consensus statements and guidelines drawing on both	34	4, 9, 12, 13, 18–22, 24, 25, 51, 60, 66, 67, 70, 74, 81, 84, 89–91, 93, 101, 103, 104, 113, 115, 126, 132–134, 140, 143
**X**	Preclinical: animal models, iPSC-derived organoids, microfluidic devices, cell lines	40	14, 23, 29–34, 36–45, 50, 52, 102, 105–112, 114, 116, 117, 119–125, 145
**–**	Methodological or reporting standard; no population class applies	2	17, 75

iPSC, induced pluripotent stem cell.
